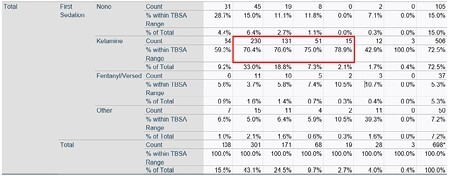# 581 Ketamine Use for Initial Sedation: A Consensus Statement from the Pediatric Injury Quality Improvement Collaborative

**DOI:** 10.1093/jbcr/irae036.215

**Published:** 2024-04-17

**Authors:** Lisa C Vitale, Mariah Malaniak, Justin D Klein, Sheila Sheila A Giles, Kyle Horvath, Rajan K Thakkar, Pediatric Surgeon, Daniel J Marx, Debra Skultety-Robinson

**Affiliations:** Children's Hospital of Michigan, Detroit, MI; Nationwide Children's Hospital, Columbus, OH; Children's Mercy Kansas City, Kansas City, MO; Johns Hopkins Children's Hospital, Baltimore, MD; Children's Hospital of Michigan, Detroit, MI; Nationwide Children's Hospital, Columbus, OH; Children's Mercy Kansas City, Kansas City, MO; Johns Hopkins Children's Hospital, Baltimore, MD; Children's Hospital of Michigan, Detroit, MI; Nationwide Children's Hospital, Columbus, OH; Children's Mercy Kansas City, Kansas City, MO; Johns Hopkins Children's Hospital, Baltimore, MD; Children's Hospital of Michigan, Detroit, MI; Nationwide Children's Hospital, Columbus, OH; Children's Mercy Kansas City, Kansas City, MO; Johns Hopkins Children's Hospital, Baltimore, MD; Children's Hospital of Michigan, Detroit, MI; Nationwide Children's Hospital, Columbus, OH; Children's Mercy Kansas City, Kansas City, MO; Johns Hopkins Children's Hospital, Baltimore, MD; Children's Hospital of Michigan, Detroit, MI; Nationwide Children's Hospital, Columbus, OH; Children's Mercy Kansas City, Kansas City, MO; Johns Hopkins Children's Hospital, Baltimore, MD; Children's Hospital of Michigan, Detroit, MI; Nationwide Children's Hospital, Columbus, OH; Children's Mercy Kansas City, Kansas City, MO; Johns Hopkins Children's Hospital, Baltimore, MD; Children's Hospital of Michigan, Detroit, MI; Nationwide Children's Hospital, Columbus, OH; Children's Mercy Kansas City, Kansas City, MO; Johns Hopkins Children's Hospital, Baltimore, MD; Children's Hospital of Michigan, Detroit, MI; Nationwide Children's Hospital, Columbus, OH; Children's Mercy Kansas City, Kansas City, MO; Johns Hopkins Children's Hospital, Baltimore, MD

## Abstract

**Introduction:**

The importance of adequate pain management for children during burn debridement is well recognized. Safe and effective procedural sedation is a fundamental quality metric for patient care in pediatric emergency departments (ED). Current literature suggests that a relatively low percentage of pediatric patients receive adequate analgesia and sedation in ED settings. The Pediatric Injury Quality Improvement Collaborative (PIQIC), an established network of five pediatric trauma and burn centers, benchmarks burn data to improve patient care and evaluate outcomes.

**Methods:**

Data was obtained across the five centers between March 2022 to August 2023 from the PIQIC database. A crosstab analysis was utilized to compare the total body surface area (TBSA) of burn injury to medication selection for sedation during initial burn debridement in the ED. A total of 698 patients (n=698) met the inclusion criteria of presenting to ED with subsequent need of admission to the burn center.

**Results:**

Among the 698 patients, the most selected sedative used amongst the 5 centers for burn debridement was ketamine. Patients with 0-1% TBSA received ketamine 59.3% of the time, respectively 1.1-5% was used 76.4%, 5.1-10% was used 76.6%, 10.1-15% was used 75%, 15.1-20% was used 78.9%, and 20.1-99% was used 42.9% of the time.

**Conclusions:**

The care of the pediatric burn patient is complex, and inadequate sedation for debridement can lead to acute and long-term psychological sequelae. Inappropriate initial sedation has also been shown to make subsequent dressing changes more difficult. Intravenous ketamine sedation provides safe and effective sedation that allows the health care team to complete adequate debridement, dressing application, and caregiver education. This data from 5 pediatric burn centers suggests that the use of intravenous ketamine sedation should be considered more broadly amongst other burn centers that care for pediatric patients.

**Applicability of Research to Practice:**

Further evaluation with prospective case controlled studies are needed to standardize this further.